# Miscellaneous

**Published:** 1888-12

**Authors:** 


### MISCELLANEOUS.

Test for Purity of Olive Oil.—To detect cotton-seed oil in olive oil, use a solution of nitrate of mercury, U. S. P. When mixed with pure olive oil the oil will become hard and solid, but if cotton-seed oil is present, the result will be a pasty, soft mass ; if it is all cotton-seed oil, it will remain fluid.

Good for Evil.—A strange scene occurred the other day at Sierck on the Moselle. Herr Schmidt had a dog which he wished to get rid of. Rowing out into the middle of the river, he fastened a stone round the dog's head and threw him into the water. The animal sank at once ; but during his struggles the rope slipped the stone, and he again rose to the surface, and tried to get back into the boat. His master, however, continued to push him back, but as the dog persevered, he lost his patience, and striking at him with his oar, lost his footing and fell into the water himself. He was unable to swim, but the dog seizing him by his coat, succeeded in bringing him to the land, after having been washed away by the the current. The dog's life was spared, we are happy to say.

A Sand Bag for a Sick Room.—Many persons are acquainted with the virtues of the hot water bag, but a sand bag is still better.

Get some clean, fine sand, dry it thoroughly in a kettle on the stove ; make a bag about eight inches square of flannel, fill it with the dry sand, sew the opening carefully together, and cover the bag with cotton or linen cloth. This will prevent the sand from sifting out, and also enable you to heat the bag quickly by placing it in the oven or on top of the stove. After once using this you will never again attempt to warm the feet or hands of a sick person with a bottle or a brick. The sand holds the heat for a long time, and the bag can be tucked up to the back without hurting the invalid.

To Purify a Room.—Set a pitcher of water in the apartment, and in a few hours it will have absorbed nearly all the respired gases in the room, the air of which will have become purer, but the water utterly filthy. The colder the water the greater the capacity to contain these gases. At the ordinary temperature a pail of water will absorb a pint of carbonic acid gas and several pints of ammonia. The capacity is nearly doubled by reducing the water to the temperature of ice. Hence the water kept in a room for awhile is unfit to use.

The common practice of raising fainting persons to a sitting or upright position is often sufficient to destroy the spark of life which remains. The death of an eminent English statesman a short time ago gave opportunity to the coroner for emphasizing this fact, and of pointing out how much more reasonable and sound it is to keep such persons in the prone position, while restoratives and local means are adopted to enable them, if possible, to regain consciousness.

Test for Lard Adulterations.—The United States Agricultural Department has discovered a simple test by which any shop keeper can detect adulteration in lard. The experiment is as follows : As much lard as can be taken up on the point of a pocket knife is placed in a tea cup. About a quarter of an ounce of sulphuric acid is placed upon it, and the two substances mixed. If the lard is pure it will coagulate, and there will be a little difficuly in uniting it with the acid. If it is adulterated with cotton seed oil or stearine, the mixture will take place immediately and easily. The substance is then poured into a common test tube. The acid, somewhat colored, will sink to the bottom, while the fatty matter will remain on top. If the lard thus tested was pure, its color will be light sponge, changing slowly to a dark cinnamon tint. If it has been adulterated with cotton seed oil the color at first will be darker, changing immediately to deep brown. The differences in the hues are so marked that a mistake cannot be made in the analysis.

HAND GRENADES.—The chemical hand grenade, which is one of the most effective devices for extinguishing fires, is as cheap to make as it is efficient in its action. For thinly populated districts, where no fire brigade is to be found within easy access, this terror of the fire-fiend should always be on hand. Thin glass bottles to hold about a quart, which can be thrown into the flames and easily broken, should be used, and the bottles should be filled with a liquid composed of ingredients, of which the following are the proper proportionate quantities, and then sealed up: 20 lbs. of common salt; 10 lbs of sal ammoniac; 7 gallons of water. If these directions are followed out the hand grenades produced will be found to be equal to any that are now being made and extensively sold throughout the country at high prices.

PROF. BAIRD says that as a fish has no maturity there is nothing to prevent it from living indefinitely and growing continually. He cites in proof a pike living in Russia whose age dated back to the fifteenth century. In the royal aquarium at St. Petersburg there are fish that have been there 140 years.—*Detroit Tribune.*

### AB AUSTRO.

A sweet wind came from out the south,
The soft south wind; but would not stay,
It scattered the breath of flowers and spice
And went away.

A bright bird flew up from the south,
With scarlet wing and breast of gray,
It roused sweet mem'ries by its song,
Then flew away.

My love came to me from the south,
Her dark eyes stole my heart away,
She brought again my vanished youth,
Just for a day.

LORING. G. BARRY.

